# The complete chloroplast genome of *Atractylodes koreana* (Nakai) Kitam and its phylogenetic analysis

**DOI:** 10.1080/23802359.2021.1928561

**Published:** 2021-06-21

**Authors:** Hongbo Xie, Mengmeng Shi, Linchun Shi, Jinxin Liu, Chunying Zhao

**Affiliations:** a Hebei Key Laboratory of Study and Exploitation of Chinese Medicine, Chengde Medical University, Chengde, China; b Institute of Medicinal Plant Development, Chinese Academy of Medical Sciences & Peking Union Medical College, Beijing, China

**Keywords:** *Atractylodes koreana*, medicinal plant, chloroplast genome, Asteraceae, phylogenetic relationship

## Abstract

*Atractylodes koreana* (Nakai) Kitam is a perennial herb of Asteraceae, mainly distributed in China and Korea, which is the main adulterant of traditional herbal medicine ‘Cangzhu’. In the present study, we reported the complete chloroplast (cp) genome of *A. koreana* with the total length of 153,232 bp, which is consisted of four regions, including one large single copy (LSC) region of 84,250 bp, one small single copy (SSC) region of 18,690 bp, and two inverted repeat regions (IRa and IRb) of 25,146 bp. The GC content of the complete cp genome is 37.7%. A total of 110 unique genes were annotated, comprising 79 protein-coding genes, 27 transfer RNA (tRNA) genes and four ribosome RNA (rRNA) genes. Moreover, nine protein-coding genes contained one intron and three protein-coding genes (*clpP*, *ycf3*, and *rps12*) contained two introns. The phylogenetic analysis indicated that *A. koreana* is a sister group of *A. chinensis* and *A. lancea*.

*Atractylodes* (Asteraceae, Cardueae) is an important medicinal plant genus endemic to East Asia and comprises approximately seven species, five of which are distributed in China (Linrong 1987). The crude drugs derived from the rhizomes of *Atractylodes* plants have been used for treating gastroduodenal and code related diseases in China, Japan, and Korea, with a medicinal history of more than 2000 years (Peng et al. 2012). Among them, the dried rhizomes of *Atractylodes lancea* (Thunb.) DC. and *Atractylodes chinensis* (Bunge) Koidz. have been categorized as ‘Cangzhu (So-jutsu, in Japanese)’, and the dried rhizome of *Atractylodes macrocephala* Koidz. has been categorized as ‘Baizhu (Byaku-jutsu, in Japanese)’ (Chinese Pharmacopoeia Commission 2020; Shiba et al. 2006). In addition, there are several easily confused species of the *Atractylodes* genus that have been used as the adulterants of ‘Cangzhu’ in the northeast China, such as *Atractylodes koreana* (Nakai) Kitam (Linrong 1987). However, the primary components obtained from *A. lancea* and *A. koreana* are significantly different, importantly the contents of atractylon and atractylodin (Liu et al. 2016). In this study, we characterized the structure of the complete chloroplast (cp) genome of *A. koreana* and analyzed its phylogenetic relationship with other species in *Atractylodes* genus.

The fresh leaves of *A. koreana* were collected from Chengde City, Hebei Province (N41°1′2″, E117°57′2″), and its plant materials (Sample ID: CDY-B-01) and voucher specimen (voucher number: HPAB008) were deposited in Chengde Medical University (http://www.cdmc.edu.cn/). The leaves of this specimen are undivided, lower and middle cauline leaves are narrowly elliptic to ovate-lanceolate, and base leaves are rounded and typically semiamplexicaul. Total genomic DNA was prepared from leaves using the plant genomic DNA extraction kit [Tiangen Biochemical Technology (Beijing) Co., Ltd, China]. The concentration and quality of extracted DNA were examined using Qubit 4.0 (Thermo Fisher Scientific, Inc., USA). The Illumina NovaSeq platform was employed for high-throughput sequencing after the genomic DNA paired-end library was constructed using sheared fragments with an average insert size of 270 bp. Approximately 2.1 GB of raw data with paired-end reads of 150 bp were generated. Trimmonmatic v0.38 was employed to remove the adapters and low-quality reads from the sequencing output files (Bolger et al. 2014). After the quality control process, the clean reads were assembled into the cp genome sequence of *A. koreana* using a NOVOPlasty toolkit (Dierckxsens et al. 2017). The gene map and unique genes of the cp genome of *A. koreana* was generated simultaneously during the annotation process using CPGAVAS2 web service (Shi et al. 2019).

The whole cp genome sequence of *A. koreana* is with a length of 153,232 bp, and its sequence has been submitted to GenBank database (accession number: MW301113). The whole cp genome of *A. koreana* is quite conserved in gene content and shows a typical circular quadripartite structure including one LSC region of 84,250 bp, one SSC region of 18,690 bp, and two inverted repeat regions (IRa and IRb) of 25,146 bp. The total GC content of the whole cp genome is 37.7%. There are 110 unique genes in the whole cp genome sequence of *A. koreana*, including 79 protein-coding genes, 27 tRNA genes and four rRNA genes (*rrn16S*, *rrn23S*, *rrn4.5S*, and *rrn5S*). Among them, nine protein-coding genes contained one intron and three protein-coding genes (*clpP*, *ycf3*, and *rps12*) contained two introns. In addition, three genes *petB*, *petD*, and *rpl16* contained small exons in the whole cp genome sequence of *A. koreana*, and the length of their small exons are 6 bp, 8 bp, and 9 bp, respectively. Moreover, *rps12* was annotated as a trans-splicing gene. In addition, there is another chloroplast genome sequence of the *A. koreana* was found in GenBank with the accession number of MT834521, whose *ycf15* gene was failed to be annotated, and four genes named *atpB*, *ndhD*, *rpoA*, and *rpoC2* were incorrectly annotated with wrong start or stop positions. Moreover, there are some single nucleotide polymorphism (SNP) sites that have been found in 17 protein-coding genes including *ccsA*, *clpP*, *ndhA*, *ndhF*, *petB*, *psaA*, *psbB*, *psbE*, *rpl16*, *rpl20*, *rpoC1*, *rps3*, *rps4*, *rps16*, *rps19*, *ycf2*, and *ycf3*. Their differences can be found in supplementary table 1.

The phylogenetic tree was constructed based on maximum likelihood (ML) method to confirm the phylogenetic position of *A. koreana* in the *Atractylodes* genus of family Asteraceae using RAxML v8.2.12 software (Stamatakis 2014) with 1000 bootstrap replicates. A total of 32 cp genomes were downloaded from GenBank for phylogenetic analysis (Cai et al. 2020; Wang et al. 2021). *Lapsanastrum humile* (Thunb.) Pak & K.Bremer from tribe Cichorieae and *Gerbera jamesonii* Bolus from tribe Mutisieae were used as the outgroup species. The phylogenetic tree showed that four species of genus *Atractylodes* formed one monophyletic clade with a high bootstrap value of 100 (Figure 1). Compared with other species in the genus *Atractylodes*, *A. koreana* independently formed a branch as the sister group of the subclade of *A. chinensis* and *A. lancea*, with the high bootstrap score. *A. koreana* is mainly distributed in Liaodong, the northern part of Shandong and Hebei provinces of China, and its lower and middle cauline leaves are undivided, which are different from the morphological characters of the other *Atractylodes* species (Zhengyi 2011). This study provides valuable genetic information for the phylogenetic relationship, taxonomic treatment, genetic diversity and identification of *A. koreana* in *Atractylodes* genus.

**Figure 1.**
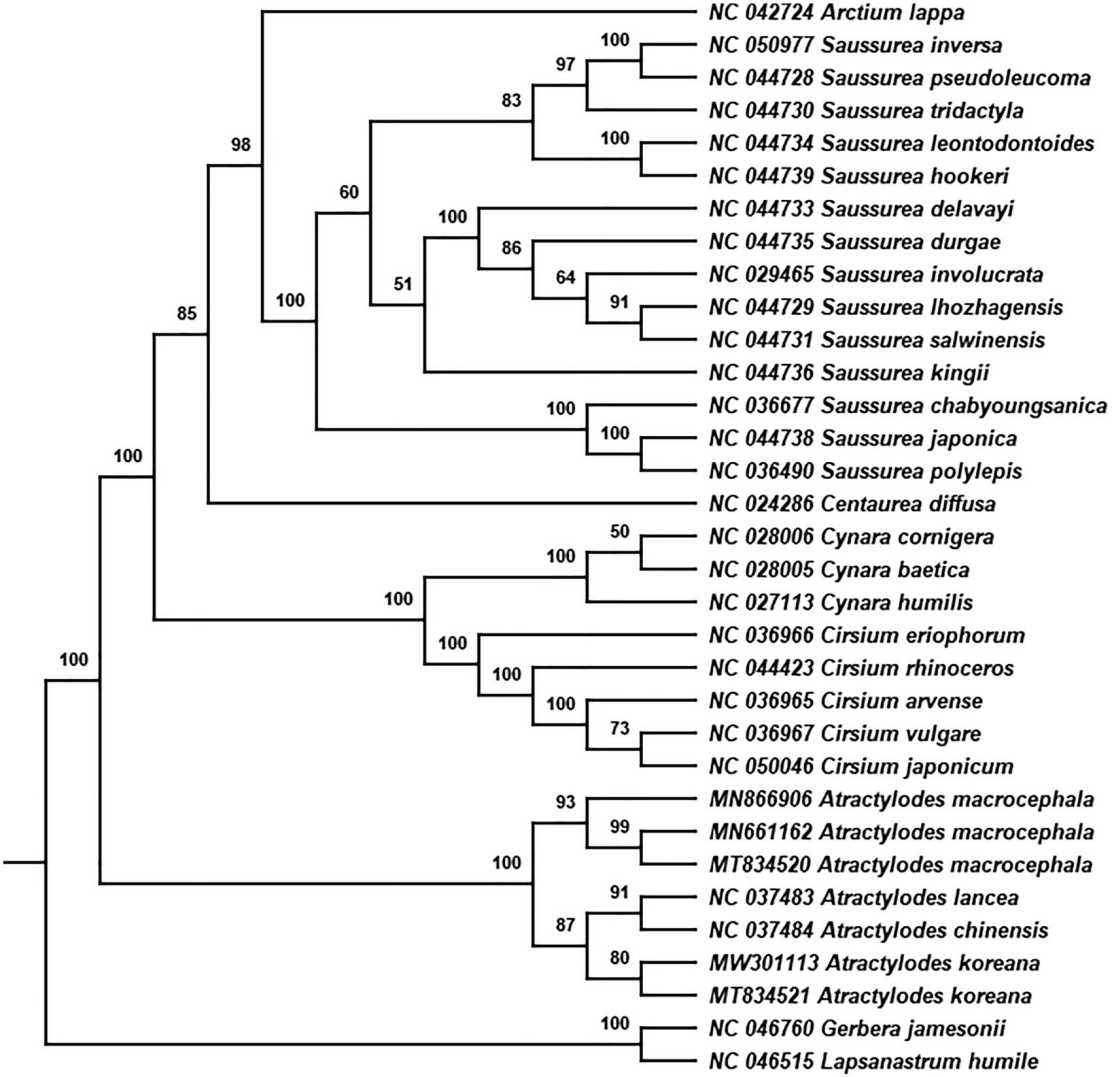
Phylogenetic tree constructed using ML method based on 33 complete cp genomes. ML bootstrap values are labeled in the corresponding branch.

## Data Availability

The genome sequence data that support the findings of this study are openly available in GenBank of NCBI at (https://www.ncbi.nlm.nih.gov/) under the accession no. MW301113. The associated Bio-Project, SRA, and Bio-Sample numbers are PRJNA682118, SRR13188655, and SAMN16985723, respectively.
